# Safety behaviors maintain persecutory ideation in individuals with psychotic disorders: evidence from an ecological momentary assessment study

**DOI:** 10.1017/S0033291726104681

**Published:** 2026-06-03

**Authors:** Sven Niklas Schönig, Tania Marie Lincoln, Katarina Krkovic

**Affiliations:** 1 Clinical Psychology & Psychotherapy, Universität Hamburg, Hamburg, Germany; 2 Clinical Psychology and Psychotherapy for Children and Youth, Universität Potsdam, Germany

**Keywords:** experience sampling method, persecutory delusions, safety-seeking behaviors, schizophrenia

## Abstract

**Background:**

Safety behaviors are a common response to persecutory ideation in psychosis. They potentially contribute to the maintenance and exacerbation of symptoms and emotional distress and are therefore a promising treatment target. However, empirical evidence for this hypothesized maintenance role is scarce. We therefore examined cross-sectional and micro-longitudinal associations between safety behaviors, negative affect, and persecutory ideation in the daily lives of individuals with psychotic disorders.

**Methods:**

We assessed safety behaviors, persecutory ideation, and negative affect at baseline and 10 times/day during one week of Ecological Momentary Assessment (EMA) in *N* = 64 participants with persecutory ideation and a psychotic disorder.

**Results:**

At baseline, safety behaviors were positively correlated with persecutory ideation (*r* = .47), depression (*r* = .67), and anxiety (*r* = .69). Safety behaviors assessed at baseline and safety behaviors assessed in daily life showed a moderate correlation (*r* = .56). Multilevel analyses of the longitudinal EMA data revealed persecutory ideation (*β* = .16) and negative affect (*β* = .11) to predict subsequent use of safety behaviors. Momentary use of safety behaviors predicted subsequent persecutory ideation (*β* = .13) and negative affect (*β* = .18).

**Conclusions:**

Our results provide evidence for a vicious cycle of maintenance involving persecutory ideation, negative affect, and safety behaviors. Targeting safety behaviors with ecological momentary interventions in daily life could be a promising approach to interrupt this cycle.

## Introduction

Feeling safe in everyday life is a fundamental need that can become compromised when people experience persecutory delusions (Bond et al., 2022; Campbell & Morrison, 2007). Persecutory delusions are associated with emotional distress (Ben-Zeev, Ellington, Swendsen, & Granholm, 2011; Krkovic, Clamor, Schlier, & Lincoln, 2020) and may elicit a variety of cognitive and behavioral responses such as conscious self-regulation attempts, threat monitoring, or reassurance seeking (Tully, Wells, Pyle, et al., 2017). Research indicates that most people experiencing persecutory delusions attempt to protect themselves against perceived threats by using *safety behaviors*: actions that aim to prevent the assumed harm from occurring (Freeman, Garety, & Kuipers, 2001; Freeman et al., 2007). While persecutory ideation represents beliefs about threat, safety behaviors represent the protective behavioral response to these beliefs. They can manifest in different ways, the most common being avoidance (e.g. not going outside due to fear of persecution) and in-situation safety behaviors (e.g. scanning one’s surroundings for persecutors; Freeman et al., 2001, 2007).

Cognitive models propose that safety behaviors are not only responses to psychotic symptoms and associated emotional distress but also implicated in their exacerbation and maintenance (Beck, Rector, Stolar, & Grant, 2008; Freeman, 2016; Morrison, 2001; Newman-Taylor & Stopa, 2013), corresponding to their established role in maintaining anxiety disorders (Clark & Wells, 1995; Helbig-Lang & Petermann, 2010; McManus, Sacadura, & Clark, 2008; Salkovskis, 1991). According to these accounts, safety behaviors prevent the disconfirmation of threat beliefs, thereby contributing to their maintenance. However, there is little causal evidence to support this assumed maintenance role in psychosis. One small experimental study (*N* = 30) compared the effects of dropping versus using safety behaviors in a virtual reality setting and found a reduction in patients’ delusional conviction and distress, thus supporting the maintenance hypothesis (Freeman et al., 2016). Most research in this field, however, has used cross-sectional designs, which do not allow causal inference. A meta-analysis of correlational studies (*k* ≤ 6) found safety behaviors to be moderately associated with persecutory ideation and weakly to moderately associated with emotional distress (i.e. anxiety, depression, and symptom-related distress) in psychosis (Tully, Wells, & Morrison, 2017). Furthermore, a recent cross-sectional study found safety behaviors to be the strongest predictor of persecutory ideation in the general population (Freeman & Loe, 2023).

As temporal precedence is a key component of causality, examining temporal processes is crucial to establishing a causal role of safety behaviors in maintaining persecutory ideation (Tully, Wells, & Morrison, 2017). Yet, little is known about the immediate temporal relationship between persecutory ideation, emotional distress, and safety behaviors in everyday life. Since safety behaviors are assumed to be a response to psychotic symptoms or emotional distress (Tully, Wells, Pyle, et al., 2017), a close temporal association with persecutory ideation and negative affect can be expected. Furthermore, if safety behaviors serve to maintain persecutory ideation, as posited in theoretical models, they should predict the subsequent occurrence thereof. Ecological Momentary Assessment (EMA) enables the investigation of such temporal associations by collecting micro-longitudinal time-series data on behaviors, symptoms, and affective states in everyday life (Oorschot, Kwapil, Delespaul, & Myin-Germeys, 2009). However, to date, the association of safety behaviors and persecutory ideation has rarely been investigated with EMA (Lüdtke, Hedelt, & Westermann, 2023). One study examined social withdrawal as a potential safety behavior in psychosis patients, relatives, and controls and found an association with concurrent persecutory ideation, but no time-lagged associations (Fett, Hanssen, Eemers, Peters, & Shergill, 2022). Another study found evidence that behavioral change in response to persecutory threat predicted negative affect and persecutory ideation the following day (Buck et al., 2024). Yet, no study has examined a wider spectrum of safety behaviors in relation to persecutory ideation and negative affect in daily life. Considering these limitations, a more comprehensive exploration of safety behaviors in everyday life is warranted.

In our study, we aimed to elucidate the potential role of safety behaviors in maintaining persecutory ideation. We investigated cross-sectional associations between safety behaviors, negative affect, and persecutory ideation as well as moment-to-moment associations during the daily lives of individuals with psychosis. We hypothesized that (1) safety behaviors at baseline would be positively associated with persecutory ideation, anxiety, and depression at baseline. Furthermore, we expected (2) a positive association between safety behaviors assessed at baseline and safety behaviors assessed in daily life. We expected that (3) persecutory ideation and (4) negative affect in daily life (*t*_i_) would predict subsequent use of safety behaviors (*t*_i + 1_) as a response. Lastly, based on the assumption that safety behaviors serve to maintain persecutory ideation, we hypothesized that (5) safety behaviors in daily life (*t*_i_) would predict subsequent persecutory ideation (*t*_i + 1_).

## Methods

### Participants

We recruited participants with schizophrenia spectrum disorders via flyers in psychiatric hospitals, outpatient clinics, and ambulatory social services in Hamburg, Germany, as well as via online announcements. Data were collected between October 2021 and July 2024. Participants were eligible if they met the following inclusion criteria: (1) age 18–65, (2) ability to provide informed consent, (3) sufficient German language skills, (4) diagnosis of a schizophrenia spectrum disorder according to the Diagnostic and Statistical Manual of Mental Disorders, Fifth Edition (DSM-5; American Psychiatric Association, 2013), and (5) a score ≥ 6 in the persecution subscale (Part B) of the Revised Green et al. Paranoid Thought Scale (R-GPTS; Freeman, Loe, Kingdon, et al., 2021), indicating at least ‘elevated persecutory ideation’ (Freeman, Loe, Kingdon, et al., 2021). This cut-off score ensured that all participants experienced persecutory ideation to a certain extent while the low threshold for inclusion also secured a wide spectrum of symptom severity. Acute suicidality, a diagnosis of dementia, severe neurological disorder, bipolar disorder, or recent substance use disorder (last substance use less than six months ago) led to exclusion (We preregistered a minimal remission interval of twelve months but lowered it to six months due to recruitment difficulties).

We calculated a required sample size of *N* = 55 with the R package PowerAnalysisIL (Lafit et al., 2021) based on unpublished data of an EMA study that examined time-lagged associations of negative affect and paranoia. We added a 15% margin of error to compensate for the imprecision of input parameters, resulting in a target sample size of *N* = 64.

### Procedure and design

The study was approved by the local ethics committee, and our hypotheses and procedure were preregistered prior to data analysis (https://osf.io/8tchn). Participants were screened for eligibility via telephone and then invited for further assessments. After giving informed consent, participants completed the R-GPTS, and a trained interviewer conducted the Structured Clinical Interview for DSM-5 sections A-D (SCID-5-CV; Beesdo-Baum et al., 2019) to assess their diagnoses. If eligible, participants were interviewed for their habitual use of safety behaviors and completed baseline symptom questionnaires and a sociodemographic questionnaire. Participants then received a smartphone with the movisensXS survey app (movisens GmbH, Karlsruhe) installed, instructions on how to respond to prompts, and a training prompt. They were instructed to respond to each prompt, if possible, and to postpone or ignore prompts only in situations in which responding would be dangerous (e.g. when driving a car) or inappropriate (e.g. during doctor’s appointments). The smartphones triggered ten EMA prompts per day between 9 AM and 10 PM for one week. Prompts occurred semi-randomly with a minimum interval of 30 minutes. After one week, participants returned the smartphone, filled out a short EMA debriefing questionnaire, and were thanked and compensated (50€).

## Measures

### Baseline measures

Persecutory ideation at baseline was assessed with the R-GPTS Part B subscale. The R-GPTS is an 18-item instrument that assesses two dimensions of paranoid ideation (Part A and B) with a 5-point Likert scale from 0: ‘not at all’ to 4: ‘totally’. Part A of the R-GPTS assesses ideas of reference (8 items, e.g.: ‘People definitely laughed at me behind my back.’) and Part B assesses ideas of persecution (10 items, e.g.: ‘I was convinced there was a conspiracy against me.’). We used the persecution subscale (Part B) as an inclusion criterion (cut-off ≥ 6; Freeman, Loe, Kingdon, et al., 2021) and as a correlate of baseline variables, as we were primarily interested in the persecutory dimension of paranoid ideation. Both subscales demonstrated good internal consistency in our sample (*α*_reference_ = .87; *α*_persecution_ = .90).

Safety behaviors were measured with the Safety Behaviors Questionnaire (SBQ; Freeman et al., 2001), a semi-structured interview that assesses the frequency of safety behavior use in the last month across seven categories (avoidance, in-situation, escape, compliance, help-seeking, aggression, delusional actions). The avoidance subscale assessed avoidance of everyday life situations, such as going outdoors or being on public transport. Behaviors used to protect oneself in distressing situations, such as reducing one’s visibility or carrying a weapon, were categorized as in-situation safety behaviors. The escape subscale assessed attempts to flee from situations perceived as threatening, and the compliance subscale comprised behaviors such as being friendly to suspected persecutors to gain their sympathy. Attempts to seek help from others (e.g. calling friends or the police) were assessed with the help-seeking subscale, and confronting suspected persecutors was assessed with the aggression subscale. Lastly, behaviors that did not logically reduce threat (e.g. thinking of going to place A despite going to place B to mislead persecutors who may be able to read one’s mind) were categorized in the ‘delusional’ subscale. The frequency of safety behaviors was rated on the adapted five-point scale by Gaynor, Ward, Garety, and Peters (2013), which adds one point to the original four-point scale by Freeman et al. (2001) to allow for more specificity. The values were then added up to subscale scores and a total score. The SBQ has demonstrated good retest reliability in its original validation study (Freeman et al., 2001). As no validated German translation of the SBQ exists, the instrument was translated into German by a native English speaker.

Depression at baseline was measured with the Beck Depression Inventory (BDI-II; Hautzinger et al., 2009), a 21-item questionnaire that assesses the severity of depression symptoms during the previous two weeks. The BDI-II has demonstrated adequate psychometric properties in psychosis populations (Scholes & Martin, 2013) and good internal consistency in our sample (*α* = .89).

Anxiety was measured with the Beck Anxiety Inventory (BAI; Margraf & Ehlers, 2007), which assesses the severity of 21 anxiety symptoms during the last week. The BAI has demonstrated adequate psychometric properties in psychosis populations (Smith, Garety, Harding, & Hardy, 2021) and good internal consistency in our sample (*α* = .89).

Negative symptoms were measured with the Motivation and Pleasure Scale – Self-Report (MAP-SR; Engel & Lincoln, 2016), a 15-item questionnaire that assesses anhedonia, social withdrawal, and avolition. Negative symptoms, such as social withdrawal, may resemble safety-seeking avoidance on the surface, which is why we used the MAP-SR to control for the shared variance that may have been captured by the SBQ. The MAP-SR has been validated in a psychosis sample (Engel & Lincoln, 2016), and Cronbach’s *α* indicated good internal consistency in our sample (*α* = .85).

### EMA measures

Persecutory ideation in daily life was measured with the Brief State Paranoia Checklist (PCL-5; Schlier, Moritz, & Lincoln, 2016), a validated five-item version of the original Paranoia Checklist (Freeman et al., 2005) that has been used in previous EMA studies (Bahlinger, Lincoln, & Clamor, 2022; Krkovic et al., 2020). Participants rated how much the item statements (e.g. ‘Shortly before the beep, people laughed at me.’) applied to them on an 11-point Likert scale (0: ‘not at all’, 10: ‘very much’). The PCL-5 demonstrated good reliability in our sample (*α_between_* = .97; *α_within_* = .77).

Negative affect in daily life was measured with four items that assessed momentary fear, sadness, anger, and shame on an 11-point Likert scale from 0: ‘not at all’ to 10: ‘very much’ (e.g. ‘Shortly before the beep, I felt anxious/fearful/scared/afraid’). This scale has been used in previous EMA studies (Krkovic, Krink, & Lincoln, 2018, Krkovic et al., 2020) and demonstrated good reliability in our sample (*α_between_* = .90; *α_within_* = .70).

Safety behaviors in daily life were measured with the Momentary Safety Behaviors Scale (MSB), a newly developed 14-item instrument to assess the momentary use of safety behaviors (Supplementary Material 1). We constructed items based on common safety behaviors named in the SBQ, as well as by consulting clinical psychologists working with psychosis patients in the local outpatient center. All items of the MSB are preceded by ‘To protect myself against other people, danger, or threat, …’ and then describe a safety behavior (e.g. ‘…I carefully observed my surroundings’.), relating to the period since the previous EMA time point. Items are rated on a scale of 0: ‘not at all’ to 6: ‘very much’ and summed up to a total score. The MSB had excellent reliability on the between-subjects-level (*α_between_* = .96) and good reliability on the within-subjects-level (*α_within_* = .78). As the MSB has not been tested for validity, we calculated the correlation between the MSB person mean and the SBQ total score as an indication of convergent validity. Further, we constructed *post hoc* subscales for the MSB by grouping items with similar content (see Supplementary Material 1) to test whether subtypes of safety behaviors assessed in daily life were correlated with the respective subtypes assessed with the SBQ.

### Data analysis

Within- and between-subjects reliability of the EMA scales was calculated with the R Shiny ‘Within-Person Research Web App’ (Yang, Wang, Huang, & Nguyen, 2022). Baseline associations were calculated with Pearson correlations. To remove the shared variance of safety behaviors and negative symptoms from these associations, we also calculated partial correlations controlling for negative symptoms (MAP-SR score).

For all hypotheses regarding moment-to-moment associations, we performed 2-level multilevel regression analyses with IBM SPSS version 29. Analyses were conducted with random intercepts and fixed slopes, as our focus was on the average within-person lagged associations rather than on the individual differences in this association. We used restricted maximum likelihood estimation and person-mean-centered predictors. For all lagged variables, we set the first value for each participant and day to ‘missing’ to avoid lagging values across participants and/or days (Viechtbauer, 2022). To facilitate comparisons between EMA variables, we transformed the MSB scores to the same 11-point rating scale as the persecutory ideation and negative affect measures. We performed sensitivity analyses to test the robustness of our results across different analysis strategies. First, we repeated our time-lagged analysis, adding the dependent variable at *t*_i_ as a predictor to control for autocorrelation. Second, we added the time point *i* as a fixed and random predictor to control for linear trends which could occur if, for example, participants became more aware of their safety behaviors during the EMA period, thereby changing their response pattern. Third, as EMA data typically have a substantial amount of missing data, we compared our preregistered strategy of missing data management (listwise deletion including all participants) with a response rate-based strategy (listwise deletion including only participants with ≥ 30% completed prompts) and with multilevel multiple imputation using the R package *mitml* (Grund, Lüdtke, & Robitzsch, 2016). The imputation and analysis procedure are detailed in Supplementary Material 2. We repeated our main analysis on a subgroup of participants who had severe or very severe persecutory ideation and therefore met Freeman et al.’s criterion for ‘likely delusions’ (R-GPTS Part B ≥ 18; Freeman, Loe, Kingdon, et al., 2021) to test whether the temporal associations were robust when applying a stricter definition of persecutory ideation. Lastly, we tested whether adding random slopes to the models changed the significance pattern.

## Results

### Sample characteristics

We contacted 155 people with a self-reported schizophrenia spectrum disorder and excluded 29 with a self-reported diagnosis of a substance use disorder and recent (< 6 months) substance use, three who had no time to participate, 48 who scored below the R-GPTS Part B cut-off for elevated persecutory ideation, six who withdrew after screening, one person who was unable to consent, three who did not meet DSM-5 criteria for a schizophrenia spectrum disorder, and one who withdrew their consent after one day of participating. This resulted in a final sample size of *N* = 64. Table 1 displays sociodemographic and clinical sample characteristics. Further sample characteristics are reported in Supplementary Material 3. As our sample comprised many patients with chronic psychosis, we examined associations between illness duration and clinical variables, none of which were significant (Supplementary Material 4).

**Table 1.** Sample characteristics Table 1. long description.

	*M* (*SD*)^a^	Range
Age	38.8 (9.9)	22–60
Gender female/male/other *n (%*)	30/34/0 (46.9/53.1/0)	–
Education level^b^: low/medium/high *n (%*)	23/32/9 (35.9/50.0/14.1)	–
DSM–5 diagnosis *n (%*)		
Schizophrenia	42 (65.6)	–
Schizoaffective disorder	18 (28.1)	–
Delusional disorder	2 (3.1)	–
Brief psychotic disorder	1 (1.6)	–
Other psychotic disorder	1 (1.6)	–
Years since illness onset	17.3 (9.8)	1.5–41
Currently taking antipsychotic medication *n (%*)	55 (85.9)	–
Olanzapine dose equivalent (oral) in mg^c^	13.6 (9.1)	0.6–40
R-GPTS Part A (reference)	15.2 (7.7)	1–32
R-GPTS Part B (persecution)	18.6 (10.1)	6–40
R-GPTS Part B score distribution *n (%*)		
6–10 (elevated)	17 (26.6)	–
11–17 (moderately severe)	17 (26.6)	–
18–27 (severe)	15 (23.4)	–
≥ 28 (very severe)	15 (23.4)	–
BDI-II	20.7 (12.6)	1–59
BAI	18.3 (10.9)	0–57
MAP-SR	26.9 (9.0)	4–48

*Note:* DSM-5, Diagnostic and Statistical Manual of Mental Disorders, Fifth Edition; R-GPTS, Revised Green et al. Paranoid Thoughts Scale; BDI-II, Beck Depression Inventory – Revised; BAI, Beck Anxiety Inventory; SBQ, Safety Behaviors Questionnaire; MAP-SR, Motivation and Pleasure Scale – Self-Report.

a or *n* (*%*) if indicated otherwise.

b Education level categories are based on levels defined by the International Standard Classification of Education (ISCED-2011; UNESCO Institute for Statistics, 2012): Low = ISCED 0–2; Medium = ISCED 3–5; High = ISCED 6–8.

c Daily defined doses according to Leucht, Samara, Heres, & Davis (2016).

Table 2 shows safety behavior types based on the SBQ subscales assessed at baseline. Fifty-nine participants (92.2%) engaged in at least one safety behavior in the previous month. Avoidant safety behaviors had the highest average frequency and in-situation safety behaviors were reported by most participants (84.4%). Aggressive and ‘delusional’ safety behaviors were the least reported types. Female participants had higher safety behavior scores (*M* = 28.2, *SD* = 19.9) than male participants (*M* = 16.3, *SD* = 17.1, *t*(62) = 2.57, *d* = .64, 95% CI [0.14, 1.14]).

**Table 2.** Safety behaviors questionnaire scores Table 2. long description.

	*M* (*SD*)	Range	*n* (*%*)^a^
SBQ Total	21.8 (19.3)	0–96	59 (92.2)
Avoidance	9.2 (11.1)	0–42	48 (75.0)
In-situation	9.1 (8.5)	0–40	54 (84.4)
Escape	0.7 (1.7)	0–10	18 (28.1)
Compliance	1.0 (1.6)	0–5	23 (35.9)
Help-seeking	0.9 (1.7)	0–7	20 (31.3)
Aggression	0.4 (1.0)	0–4	13 (20.3)
Delusional	0.5 (1.7)	0–10	5 (7.8)
Number of safety behaviors	6.9 (5.2)	0–21	–
Perceived effectiveness	5.7 (3.0)	0–10	–
Perceived control	5.7 (2.9)	0–10	–

*Note*: SBQ, Safety Behaviors Questionnaire.

a *n* (*%*) with at least one reported safety behavior in the respective category.

### EMA characteristics

A total of 4082 EMA prompts were delivered to participants (*M* = 63.8, *SD* = 7.0), and 2732 were responded to (*M* = 42.7, *SD* = 17.0). The mean time interval between prompts was 78.1 minutes, and the median time to complete a prompt was 85 seconds. Participants’ mean response rate (= prompts completed/prompts delivered) was 66.6%, indicating that a third of prompts was missed or ignored. Seven participants (10.9%) had a response rate of less than 30%. Participants’ response rate showed no association with their baseline anxiety, depression, persecutory ideation, negative symptoms, safety behaviors, gender, age, or any of the debriefing questionnaire items (Supplementary Material 5).

### Associations between baseline variables (H1)

More frequent safety behavior use in the previous month was associated with more persecutory ideation, anxiety, and depression (Table 3). These correlations remained significant when negative symptoms (MAP-SR score) were controlled for (Supplementary Material 6).

**Table 3.** Correlations between baseline variables Table 3. long description.

Variable	SBQ	R-GPTS Part B	BDI-II	BAI
SBQ	–			
R-GPTS Part B	.47***	–		
BDI-II	.67***	.42**	–	
BAI	.69***	.29*	.52***	–

*Note*: SBQ, Safety Behaviors Questionnaire; R-GPTS Part B, Revised Green et al. Paranoid Thought Scale – Persecution Subscale; BDI-II, Beck Depression Inventory – Revised; BAI, Beck Anxiety Inventory. All tests are two-tailed. *p*-values are Bonferroni-Holm corrected. *p < .05 **p < .01 ***p < .001

### Associations between habitual and momentary safety behaviors (H2)

Habitual safety behaviors assessed at baseline (SBQ score) and momentary safety behaviors assessed in daily life (MSB person mean) were positively correlated (*r* = .56, *p* < .001). An exploratory analysis with *post hoc* created subscales for the MSB showed that the avoidance (*r* = .53, *p* < .001) and escape subscales (*r* = .44, *p* = .01) were positively correlated across instruments, whereas the in-situation, help-seeking, and aggression subscales were not (Supplementary Material 7).

### Temporal associations in daily Life (H3-H5)

The intraclass correlation (ICC) for safety behaviors as a dependent variable was *ρ* = .73, and the ICCs for persecutory ideation and negative affect were *ρ* = .76 and *ρ* = .62, respectively. This indicates that there was substantial between-person variance on all EMA measures, whereas within-person variance was relatively low (Heck, Thomas, & Tabata, 2013). Negative affect and persecutory ideation were correlated (*r* = .63, *p* < .001). Table 4 displays the fixed effects of the multilevel regression analysis. In line with our hypotheses, persecutory ideation at *t*_i_ and negative affect at *t*_i_ positively predicted subsequent safety behaviors at *t*_i + 1_. Also supporting our prediction, safety behaviors at *t*_i_ predicted persecutory ideation at *t*_i+1_. Lastly, safety behaviors predicted negative affect at *t*_i+1_ (Table 4).

**Table 4.** Multilevel time-lagged effects in daily life Table 4. long description.

Fixed effects	*b*(*SE*)	95%CI	*β*	*p*
Outcome: Safety behaviors *t*_i + 1_				
Intercept	2.29 (0.23)			
Persecutory ideation *t*_i_	0.15 (0.02)	[0.10; 0.19]	.16	<.001
Outcome: Safety behaviors *t*_i + 1_				
Intercept	2.29 (0.23)			
Negative affect *t*_i_	0.12 (0.02)	[0.08; 0.16]	.11	<.001
Outcome: Persecutory ideation *t*_i + 1_				
Intercept	1.49 (0.25)			
Safety behaviors *t*_i_	0.14 (0.02)	[0.09; 0.19]	.13	<.001
Outcome: Negative affect *t*_i + 1_				
Intercept	1.72 (0.19)			
Safety behaviors *t*_i_	0.16 (0.03)	[0.11; 0.22]	.18	<.001

*Note*: *b* = unstandardized coefficient. SE, standard error; CI, confidence interval; *β* = standardized coefficient. Each row segment represents a separate model. All models were calculated with fixed slopes and a random intercept.

### Sensitivity analyses

Fixed effects remained significant when the dependent variable at *t*_i_ was added as a predictor to control for autocorrelation (Supplementary Material 8), when the time point *i* was added as a predictor to control for linear time trends (Supplementary Material 9), when only participants with an EMA response rate ≥ 30% were analyzed (Supplementary Material 10), when multiple imputation was performed (Supplementary Material 11), and when only participants with severe/very severe persecutory ideation were analyzed (Supplementary Material 12). As the ICCs indicated high variability between individuals, we tested whether modeling between-person differences with random slopes would provide a better fit to the data. Adding random slopes produced the same pattern of significant results (Supplementary Material 13).

## Discussion

This study is the first to examine both cross-sectional and temporal associations between safety behaviors, persecutory ideation, and emotional distress in people with psychotic disorders. As hypothesized, habitual safety behaviors were positively associated with persecutory ideation, anxiety, and depression at baseline. Furthermore, habitual safety behaviors were associated with our measure of momentary safety behaviors in daily life, indicating that both assessed a similar construct. The analysis of time-lagged relationships in daily life revealed that persecutory ideation and negative affect predicted subsequent safety behaviors. Conversely, safety behaviors predicted subsequent persecutory ideation and negative affect, suggesting the existence of a self-perpetuating cycle involved in the maintenance and exacerbation of persecutory ideation and negative affect.

Our results support the notion that people with psychotic disorders and persecutory ideation regularly carry out safety behaviors to protect themselves against threats. In line with previous research showing a high prevalence of safety behaviors among people with persecutory delusions (Freeman et al., 2001, 2007; Gaynor et al., 2013), 92% of our sample engaged in one or more safety behaviors over the past month. Most instances of safety-seeking were attempts to avoid feared situations or to endure them with in-situation safety behaviors, consistent with earlier research investigating common types of safety behaviors in psychosis (Freeman et al., 2001, 2007; Hacker, Birchwood, Tudway, Meaden, & Amphlett, 2008). Thus, safety behaviors seem to be central to the lived experience of people with psychotic disorders and persecutory ideation.

At a cross-sectional level, we found evidence that the habitual use of safety behaviors is associated with the severity of persecutory ideation, anxiety, and depression. This is in line with meta-analytic findings linking safety behaviors to different dimensions of psychopathology (Tully, Wells, & Morrison, 2017). Our findings suggest that people with psychotic disorders who experience higher levels of distressing transdiagnostic symptoms tend to engage in safety behaviors more frequently. Controlling for negative symptoms did not affect the pattern of results, which indicates that the overlap of negative symptoms and safety behaviors was not driving the cross-sectional associations. Surprisingly, in our sample, safety behaviors were more strongly associated with depression and anxiety than previously reported (Tully, Wells, & Morrison, 2017), surpassing the association with persecutory ideation. One reason for this could be that our sample included many individuals with chronic symptoms. In chronic stages of psychosis, safety behaviors may evolve from specific threat responses into more habituated and generalized protective mechanisms. They may then serve to mitigate broader emotional distress and negative affect rather than being linked exclusively to persecutory ideation. It is also possible that individuals who frequently engage in safety behaviors experience their defenses as insufficiently effective in preventing persecutory threat, which may exacerbate helplessness (Upthegrove, Ross, Brunet, McCollum, & Jones, 2014) and result in increased depression and anxiety. Investigating the perceived effectiveness of safety behaviors in more detail could provide evidence to test this hypothesis.

Habitual and momentary safety behavior use were moderately associated, which supports the validity of the MSB total score. Exploratory analyses revealed that some safety behavior subtypes were correlated across instruments, suggesting overlap between the assessment of safety behaviors in everyday life and the assessment in the interview. Specifically, the avoidance and escape subscales were associated across instruments and settings, whereas the in-situation, aggression, and help-seeking subscales were not. While this could point to a lack of convergent validity of some of these *post hoc* constructed subscales, it could also indicate that the MSB was able to capture subtle behaviors in everyday life that were missed in the interview, which could explain the insignificant associations between some subscales.

The investigation of temporal associations in participants’ everyday lives revealed that both persecutory ideation and negative affect predicted the subsequent use of safety behaviors. This is consistent with another EMA study that found negative affect to predict next-day behavioral change in response to persecutory ideation (Buck et al., 2024), although our findings suggest that the association may occur on a shorter time scale within hours rather than days. Our data therefore show that there is a close time-lagged association between momentary experiences of threat, emotional distress, and the use of safety behaviors in psychosis. While safety behaviors may not always be a response to distress and may also become habitual over time and thus less dependent on emotional precursors, our results clearly indicate that moment-to-moment fluctuations in persecutory ideation and negative affect are related to subsequent fluctuations in safety seeking. Adding to these results, we found that the momentary use of safety behaviors predicted subsequent persecutory ideation and negative affect in participants’ everyday lives. As such, safety behaviors are not only used in response to persecutory ideation and negative affect, but they also seem to contribute to the exacerbation and maintenance of these distressing experiences. Thus, our findings support the idea that safety behaviors, persecutory ideation, and negative affect are involved in a self-perpetuating vicious cycle. This idea aligns with cognitive behavioral models that describe safety behaviors as a maintenance factor of persecutory delusions (Beck et al., 2008; Freeman, 2016; Morrison, 2001; Newman-Taylor & Stopa, 2013). Notably, although our study’s inclusion criteria only required the presence of persecutory ideation (including mild and moderate manifestations) rather than delusions, a subgroup analysis of participants with severe or very severe persecutory ideation, i.e. with ‘likely delusions’ (Freeman, Loe, Kingdon, et al., 2021), yielded the same pattern of results. Therefore, it is likely that the self-perpetuating maintenance process we identified also translates to persecutory delusions. The moment-to-moment associations observed in our study are consistent with previous findings of a similar vicious cycle of negative affect, behavioral responses, and persecutory ideation unfolding from one day to the next (Buck et al., 2024). Furthermore, our findings add to the results of another EMA study that found voice hearing-related safety behaviors to predict subsequent voice distress (Fielding-Smith, Greenwood, Wichers, Peters, & Hayward, 2022), suggesting that safety behaviors are implicated in the maintenance of other psychotic symptoms as well.

Previous theoretical work has suggested that using safety behaviors results in a short-term relief that reinforces the underlying threat belief and promotes the misattribution of safety to one’s safety behaviors (Salkovskis, 1991). At the time scale we examined in this study, safety behaviors seemed to exacerbate, rather than mitigate negative affect. Our results clearly demonstrate a detrimental effect of safety behaviors on emotional distress. However, they do not rule out the possibility of a more immediate short-term relief and investigating the hypothesized short-term functionality of safety behaviors remains an interesting avenue for future research.

From a practical clinical perspective, our results suggest that reducing safety behaviors has the potential to interrupt the maintenance of persecutory ideation and negative affect. This idea is not novel, as previous cognitive-behavioral interventions for persecutory delusions have included safety behaviors as a treatment target, e.g. in virtual reality environments (Berkhof et al., 2024; Freeman et al., 2022, 2023; Pot-Kolder et al., 2018) and face-to-face treatments (Freeman, Emsley, Diamond, et al., 2021). However, our study suggests that, in addition to these approaches, it may be beneficial to provide momentary interventions in individuals’ everyday lives to disrupt short-term maintenance processes and facilitate the transfer of behavioral change. In line with current theoretical frameworks of psychotic symptom maintenance (Sheffield et al., 2024), dropping safety behaviors in the natural context of everyday life, potentially with the help of mobile apps, may help people with psychotic disorders regain an intrinsic sense of safety and break the cycle of symptom maintenance and exacerbation.

Our findings should be interpreted in the light of several limitations. First, our data were partially collected during the COVID-19 pandemic, which may have affected our assessment of safety behaviors. Specifically, the recommendation of social distancing during this period could have amplified individuals’ tendency to self-isolate, avoid social interactions, and seek safety. Second, while our micro-longitudinal findings support the assumption that safety behaviors causally maintain persecutory ideation, only experimental designs can establish true causality. Previous research has provided first evidence for this (Freeman, 2016), but experimental studies with larger samples in more natural contexts are needed. Third, our sample included many participants with chronic psychosis. Our findings may therefore not be representative of individuals with first-episode psychosis. Although illness duration showed no association with clinical variables, which suggests limited impact of chronicity, it is possible that this was due to restricted variance as our sample included very few participants with recent illness onset. In addition, the temporal associations we found were small, which indicates that safety behaviors are one of many other factors relevant to the maintenance of persecutory ideation. The results of our time-lagged analysis hinge on the chosen time intervals of approximately 80 minutes (with random variation) and may have produced different results with another sampling schedule. Moreover, the temporal association between safety behaviors and subsequent negative affect was not included in our preregistered hypotheses and should therefore be interpreted with caution. Lastly, our study had a substantial amount of missing EMA data, which limits the conclusiveness of our results. Although the EMA response rate in our study was within the average range of other studies in psychosis samples (Bell et al., 2024), and we conducted analyses to combat missing data issues and demonstrate the robustness of our results, we cannot rule out that missingness was related to an unobserved factor.

The present study provides compelling evidence that safety behaviors play a crucial role in the maintenance of persecutory ideation and associated emotional distress in people with psychotic disorders. Safety-seeking is a common and understandable reaction to persecutory threats but seems to uphold the impression of impending harm rather than mitigate it. Reducing safety behaviors may disrupt the vicious cycle maintaining persecutory ideation and emotional distress and help people with psychotic disorders feel safer in their everyday lives.

## Supporting information

10.1017/S0033291726104681.sm001 Schönig et al. supplementary material Schönig et al. supplementary material

## Supplementary material

The supplementary material for this article can be found at http://doi.org/10.1017/S0033291726104681.

## Table 1. Long description

The table has three columns: variable, mean with standard deviation or count with percent, and range. The first row lists age with mean 38.8 years, standard deviation 9.9, range 22 to 60. Gender is 30 female, 34 male, 0 other, corresponding to 46.9 percent female, 53.1 percent male, 0 percent other. Education level is 23 low, 32 medium, 9 high, or 35.9 percent low, 50.0 percent medium, 14.1 percent high. Under D S M dash 5 diagnosis, 42 have schizophrenia (65.6 percent), 18 schizoaffective disorder (28.1 percent), 2 delusional disorder (3.1 percent), 1 brief psychotic disorder (1.6 percent), 1 other psychotic disorder (1.6 percent). Years since illness onset is mean 17.3, standard deviation 9.8, range 1.5 to 41. Currently taking antipsychotic medication: 55 individuals, 85.9 percent. Olanzapine dose equivalent (oral) mean is 13.6 milligrams, standard deviation 9.1, range 0.6 to 40. R dash G P T S Part A mean is 15.2, standard deviation 7.7, range 1 to 32. R dash G P T S Part B mean is 18.6, standard deviation 10.1, range 6 to 40. R dash G P T S Part B score distribution: 17 elevated (6 to 10, 26.6 percent), 17 moderately severe (11 to 17, 26.6 percent), 15 severe (18 to 27, 23.4 percent), 15 very severe (28 or higher, 23.4 percent). B D I dash I I mean is 20.7, standard deviation 12.6, range 1 to 59. B A I mean is 18.3, standard deviation 10.9, range 0 to 57. M A P dash S R mean is 26.9, standard deviation 9.0, range 4 to 48. Table notes define all initialisms and provide footnotes for education and medication dose equivalence.

Navigate back to Table 1..

## Table 2. Long description

The table has four rows and four columns. The first column lists: S B Q Total, Number of safety behaviors, Perceived effectiveness, and Perceived control. The second column, labeled M open parenthesis S D close parenthesis, gives means and standard deviations: 21.8 open parenthesis 19.3 close parenthesis for S B Q Total, 6.9 open parenthesis 5.2 close parenthesis for Number of safety behaviors, 5.7 open parenthesis 3.0 close parenthesis for Perceived effectiveness, and 5.7 open parenthesis 2.9 close parenthesis for Perceived control. The third column, Range, shows 0 to 96 for S B Q Total, 0 to 21 for Number of safety behaviors, and 0 to 10 for both Perceived effectiveness and Perceived control. The fourth column, n open parenthesis percent close parenthesis, lists 59 open parenthesis 92.2 close parenthesis for S B Q Total and dashes for the other rows. A note below the table defines S B Q as Safety Behaviors Questionnaire and clarifies that n open parenthesis percent close parenthesis refers to participants with at least one reported safety behavior in the respective category.

Navigate back to Table 2..

## Table 3. Long description

The table has four variables listed both as rows and columns: SBQ, R-GPTS Part B, BDI-II, and BAI. Diagonal cells contain dashes, indicating self-correlation is not applicable. Off-diagonal cells display Pearson correlation coefficients with asterisks denoting significance. From the top row, SBQ correlates with R-GPTS Part B at point four seven triple asterisk, with BDI-II at point six seven triple asterisk, and with BAI at point six nine triple asterisk. R-GPTS Part B correlates with BDI-II at point four two double asterisk and with BAI at point two nine single asterisk. BDI-II correlates with BAI at point five two triple asterisk. Asterisks indicate significance levels: single asterisk for p less than point zero five, double asterisk for p less than point zero one, triple asterisk for p less than point zero zero one. All tests are two-tailed and p-values are Bonferroni-Holm corrected. Variable definitions: SBQ is Safety Behaviors Questionnaire, R-GPTS Part B is Revised Green et al. Paranoid Thought Scale Persecution Subscale, BDI-II is Beck Depression Inventory Revised, BAI is Beck Anxiety Inventory.

Navigate back to Table 3..

## Table 4. Long description

The table is divided into four vertical segments, each anchored by an outcome variable at time i plus 1. The first segment, outcome safety behaviors at time i plus 1, lists intercept 2.29 with standard error 0.23, followed by persecutory ideation at time i with coefficient 0.15, standard error 0.02, confidence interval 0.10 to 0.19, standardized beta 0.16, and p-value less than .001. The second segment, also outcome safety behaviors at time i plus 1, lists intercept 2.29 with standard error 0.23, followed by negative affect at time i with coefficient 0.12, standard error 0.02, confidence interval 0.08 to 0.16, standardized beta 0.11, and p-value less than .001. The third segment, outcome persecutory ideation at time i plus 1, lists intercept 1.49 with standard error 0.25, followed by safety behaviors at time i with coefficient 0.14, standard error 0.02, confidence interval 0.09 to 0.19, standardized beta 0.13, and p-value less than .001. The fourth segment, outcome negative affect at time i plus 1, lists intercept 1.72 with standard error 0.19, followed by safety behaviors at time i with coefficient 0.16, standard error 0.03, confidence interval 0.11 to 0.22, standardized beta 0.18, and p-value less than .001. Each segment represents a separate model with fixed slopes and a random intercept.

Navigate back to Table 4..
