# Supplementary material for: Safety behaviors maintain persecutory ideation in individuals with psychotic disorders: evidence from an ecological momentary assessment study
Source: Psychol Med. 2026 Jun 3;56:e177. doi: 10.1017/S0033291726104681 (PMC13234519; doi:10.1017/S0033291726104681)
Supplement: Schönig et al. supplementary material [file S0033291726104681sup001.docx]

**Safety Behaviours Maintain Persecutory Ideation in Individuals with Psychotic Disorders: Evidence from an Ecological Momentary Assessment Study**

**- SUPPLEMENT -**

**Supplementary Material 1**

Momentary Safety Behaviours (MSB)

“This part addresses how you have dealt with thoughts and feelings of threat. The questionnaire below lists a few possible responses. People who feel threatened by others sometimes act in one or more of the following ways to protect themselves. For each statement, please indicate whether you have responded in this way since the last prompt.
To protect myself from other people, danger or threat, ...”

|  | 0  Not at all | 1 | 2 | 3 Some-what | 4 | 5 | 6 Very much |
| --- | --- | --- | --- | --- | --- | --- | --- |
| 1)...I avoided an activity, a place or a situation. |  |  |  |  |  |  |  |
| 2)...I avoided personal contact or eye contact with other people. |  |  |  |  |  |  |  |
| 3)...I escaped from a situation in a hurry. |  |  |  |  |  |  |  |
| 4)...I carried certain objects with me that make me feel safer. |  |  |  |  |  |  |  |
| 5)...I distracted myself |  |  |  |  |  |  |  |
| 6)...I was vigilant and alert. |  |  |  |  |  |  |  |
| 7)...I got angry or aggressive towards other people. |  |  |  |  |  |  |  |
| 8)...I tried to get help from people I know or the police. |  |  |  |  |  |  |  |
| 9)...I tried not to attract attention to myself. |  |  |  |  |  |  |  |
| 10)...I went to a safe space. |  |  |  |  |  |  |  |
| 11) ...I researched information. |  |  |  |  |  |  |  |
| 12)...I carefully observed my surroundings. |  |  |  |  |  |  |  |
| 13)...I examined everyday objects or food. |  |  |  |  |  |  |  |
| 14)...I talked to someone about my thoughts. |  |  |  |  |  |  |  |

*Note*. The following subscales were constructed *post hoc*: avoidance (items 1, 2, 10); in-situation (items 4, 5, 6, 9, 11, 12, 13); escape (item 3); getting help (items 8, 14); aggression (item 7).

**Supplementary Material 2**

We used the R package *mitml* to impute missing EMA data and *lme4* to fit linear mixed models for the imputed datasets. In *mitml*, we used the *pan* algorithm for imputation, a Markov Chain Monte Carlo (MCMC) procedure to draw replacement values for missing data. During the initial burn-in phase, no imputations are saved and the algorithm runs ‘burn-in’ iterations to reach convergence before imputed sets are drawn. Based on the models of interest [1] – [4] that were derived from our hypotheses, we specified two imputation models:

**Models of interest:**

[1] Random intercept, fixed effect of persecutory ideation at *t*_i_ on safety behaviours at *t*_i+1_

${SB}_{(i+1)j}= \beta_{0}+\beta_{1}{PERSEC}_{ij}+\upsilon_{0j}+\varepsilon_{ij}.$

[2] Random intercept, fixed effect of negative affect at *t*_i_ on safety behaviours at *t*_i+1_

${SB}_{(i+1)j}= \beta_{0}+\beta_{1}{NA}_{ij}+\upsilon_{0j}+\varepsilon_{ij}.$

[3] Random intercept, fixed effect of safety behaviours at *t*_i_ on persecutory ideation at *t*_i+1_

${PERSEC}_{(i+1)j}= \beta_{0}+\beta_{1}{SB}_{ij}+\upsilon_{0j}+\varepsilon_{ij}.$

[4] Random intercept, fixed effect of safety behaviours at *t*_i_ on negative affect at *t*_i+1_

${NA}_{(i+1)j}= \beta_{0}+\beta_{1}{SB}_{ij}+\upsilon_{0j}+\varepsilon_{ij}.$

**Imputation model for models [1] and [2]:**

formula.1 <- sb + persec_lag1 + na_lag1 ~ 1 + (1|ID)

**Imputation model for models [3] and [4]:**

formula.2 <- sb_lag1 + persec + na ~ 1 + (1|ID)

We ran *n* = 200000 burn-in iterations as convergence criteria ($\hat{R}$, visual inspection of trace plots) indicated that all parameters had reached a stationary distribution after this number of iterations. Then, *m* = 100 imputed data sets were drawn, each 5000 iterations apart, so that draws would be independent. The R code for the multiple imputation procedure can be found here: <https://osf.io/bsnvz/files/wq62j>.

**Supplementary Material 3**

**Table S-3**

*Additional Sample Characteristics*

|  | *n* (*%*) |
| --- | --- |
| Marital Status |  |
| Single | 49 (76.6) |
| Married | 3 (4.7) |
| Divorced/separated | 6 (8.4) |
| Cohabiting | 6 (8.4) |
| Net monthly income |  |
| 0 -500€ | 19 (29.7) |
| 500€ - 1000€ | 19 (29.7) |
| 1000€ - 1500€ | 15 (23.4) |
| 1500€ - 2000€ | 2 (3.1) |
| 2000€ - 2500€ | 2 (3.1) |
| 2500€ - 3000€ | 2 (3.1) |
| Employment |  |
| Unemployed | 23 (35.9) |
| Employed (part-time) | 5 (7.8) |
| Employed (full-time) | 3 (4.7) |
| Student | 3 (4.7) |
| Sheltered workshop | 6 (9.4) |
| Retired (medical) | 18 (28.1) |
| Retired (age) | 1 (1.6) |
| Undergoing education | 1 (1.6) |
| Houseman/housewife | 2 (3.1) |

| R-GPTS Part A (reference) |  |
| --- | --- |
| 0 - 9 (average) | 12 (18.8) |
| 10 - 15 (elevated) | 26 (40.6) |
| 16 - 20 (moderately severe) | 12 (18.8) |
| 21 - 24 (severe) | 6 (9.4) |
| ≥ 25 (very severe) | 8 (12.5) |
| R-GPTS Part B (persecution) |  |
| 6 - 10 (elevated) | 17 (26.6) |
| 11 - 17 (moderately severe) | 17 (26.6) |
| 18 - 27 (severe) | 15 (23.4) |
| ≥ 28 (very severe) | 15 (23.4) |
| BDI-II |  |
| 0 - 13 (minimal depression) | 20 (31.3) |
| 14 - 19 (mild depression) | 17 (26.6) |
| 20 - 28 (moderate depression) | 11 (17.2) |
| ≥ 29 (severe depression) | 16 (25.0) |
| BAI |  |
| 0 - 13 (minimal anxiety) | 8 (12.5) |
| 14 - 19 (mild anxiety) | 22 (34.4) |
| 20 - 28 (moderate anxiety) | 21 (32.8) |
| ≥ 29 (severe anxiety) | 13 (20.3) |

*Note.* R-GPTS = Revised Green et al. Paranoid Thoughts Scale; BDI-II = Beck Depression Inventory – Revised; BAI = Beck Anxiety Inventory.

**Supplementary Material 4**

**Table S-4**

*Correlations Between Illness Duration and Clinical Variables*

| Clinical variables | *r* | *p* |
| --- | --- | --- |
| MAP-SR | -.24 | .08 |
| R- GPTS_persecution_ | .09 | .52 |
| SBQ | .25 | .07 |
| BDI-II | .07 | .59 |
| BAI | .17 | .22 |

*Note*. MAP-SR = Motivation and Pleasure Scale – Self-Report; R-GPTS_persecution_ = Revised Green et al. Paranoid Thought Scale – Persecution subscale; SBQ = Safety Behaviours Questionnaire; BDI-II = Beck Depression Inventory – Revised; BAI = Beck Anxiety Inventory. All tests are two-tailed.

**Supplementary Material 5**

**Table S-5**

*Associations of EMA Response Rate With Baseline and Debriefing Variables*

| Variable | *r* | *p* |
| --- | --- | --- |
| BAI | -.06 | .64 |
| BDI-II | .05 | .72 |
| R-GPTS_reference_ | -.14 | .26 |
| R-GPTS_persecution_ | -.09 | .48 |
| MAP-SR | .14 | .26 |
| SBQ | -.00 | .99 |
| Gender | .11 | .37 |
| Age | .17 | .20 |
| DQ1^a^ | -.25 | .85 |
| DQ2 ^a^ | .08 | .55 |
| DQ3 ^a^ | -.03 | .84 |
| DQ4 ^a^ | .23 | .07 |
| DQ5 ^a^ | -.05 | .69 |
| DQ6 ^a^ | .11 | .41 |
| DQ7 ^a^ | .02 | .89 |
| DQ8 ^a^ | -.06 | .66 |
| DQ9 ^a^ | -.07 | .62 |
| DQ10 ^a^ | .13 | .32 |
| DQ11 ^a^ | .08 | .54 |
| DQ12 ^a^ | .00 | .98 |
| DQ13 ^a^ | -.05 | .71 |
| DQ14 ^a^ | -.08 | .56 |

*Note.* BAI = Beck Anxiety Inventory; BDI-II = Beck Depression Inventory – Revised; R-GPTS = Revised Green et al. Paranoid Thought Scale; MAP-SR = Motivation and Pleasure Scale – Self-Report; SBQ = Safety Behaviours Questionnaire; DQ = Debriefing Question; DQ1 = “Was the past week rather average for you? Like the time before?” DQ2 = “Were there any important/exceptional events for you in the past week?”; DQ3 = “Did others react negatively to the study smartphone?”; DQ4 = “Did others react positively to the study smartphone?”; DQ5 = “Were you uncomfortable with these reactions?”; DQ6 = “How uncomfortable was it for you to carry the smartphone?”; DQ7 = “How uncomfortable were you with the self-assessments on the smartphone?”; DQ8 = “Were you able to express your actual experience using the questions we provided?”; DQ9 = “Were the prompts too frequent?”; DQ10 = “Did you behave differently than usual in some situations as a result of the survey?”; DQ11 = “Did you wait for the prompts?”; DQ12 = “Did you pay more attention to your body than usual during the survey?”; DQ13 = “Did you pay more attention to your mental state than usual during the survey?”; DQ14 = “Did you consciously avoid situations/places/people that trigger emotions in you?”. ^a^ rated on a 5-point Likert scale from 1: “not at all” to 5: “completely”. All tests are two-tailed.

**Supplementary Material 6**

**Table S-6**

*Partial Correlations of Baseline Variables Controlling for MAP-SR Score*

| R-GPTS_persecution_ | SBQ | BDI-II | BAI |
| --- | --- | --- | --- |
| – |  |  |  |
| .44*** | – |  |  |
| .39** | .64*** | – |  |
| .28* | .70*** | .53*** | – |

*Note*. MAP-SR = Motivation and Pleasure Scale – Self-Report; R-GPTS_persecution_ = Revised Green et al. Paranoid Thought Scale – Persecution subscale; SBQ = Safety Behaviours Questionnaire; BDI-II = Beck Depression Inventory – Revised; BAI = Beck Anxiety Inventory. All tests are two-tailed. *p*-values were Bonferroni-Holm corrected.
**p* < .05 ***p* < .01 ****p* < .001

**Supplementary Material 7**

**Table S-7**

*Correlations Between Subtypes of Habitual Safety Behaviours and Momentary Safety Behaviours*

| **Habitual Safety Behaviours** | **Momentary Safety Behaviours** | | | | | |
| --- | --- | --- | --- | --- | --- | --- |
|  | Total | Avoidance | In-situation | Escape | Help-seeking | Aggression |
| Total | .56*** | .56*** | .54*** | .49** | .39* | .45** |
| Avoidance | .53*** | .53*** | .49** | .47** | .39* | .48** |
| In-situation | .36 | .40* | .34 | .29 | .20 | .17 |
| Escape | .40* | .39* | .37 | .44** | .30 | .37 |
| Compliance | .31 | .27 | .30 | .28 | .28 | .15 |
| Help-seeking | .26 | .18 | .27 | .18 | .19 | .40* |
| Aggression | .19 | .16 | .18 | .19 | .14 | .24 |
| Delusional | .05 | -.00 | .09 | -.04 | .02 | .08 |

*Note*. Habitual safety behaviours were measured with the Safety Behaviours Questionnaire (SBQ) and momentary safety behaviours were measured with the Momentary Safety Behaviours Scale (MSB). The subscales of the MSB were created *post hoc* by grouping similarly themed items. All tests are two-tailed. *p*-values were Bonferroni-Holm corrected. **p* < .05 ***p* < .01 ****p* < .001

**Supplementary Material 8**

**Table S-8**

*Multilevel Time-Lagged Effects in Daily Life Controlling for Autocorrelation*

| **Fixed Effects** | ***b*(*SE*)** | **95%CI** | ***β*** | ***p*** | |
| --- | --- | --- | --- | --- | --- |
| **Outcome: Safety behaviours *t*_i+1_** | | | | |  |
| Intercept | 2.09 (0.21) |  |  |  | |
| Persecutory ideation *t*_i_ | 0.07 (0.02) | [0.03; 0.12] | .07 | <.001 | |
| Safety behaviours *t*_i_ | 0.22 (0.02) | [0.17; 0.26] | .22 | <.001 | |
| **Outcome: Safety behaviours *t*_i+1_** | | | | | |
| Intercept | 2.08 (0.21) |  |  |  | |
| Negative affect *t*_i_ | 0.06 (0.02) | [0.02; 0.10] | .05 | .002 | |
| Safety behaviours *t*_i_ | 0.21 (0.02) | [0.18; 0.27] | .22 | <.001 | |
| **Outcome: Persecutory ideation *t*_i+1_** | | | | | |
| Intercept | 1.54 (0.26) |  |  |  | |
| Safety behaviours *t*_i_ | 0.08 (0.03) | [0.03; 0.13] | .08 | .002 | |
| Persecutory ideation *t*_i_ | 0.16 (0.02) | [0.12; 0.21] | .15 | <.001 | |
| **Outcome: Negative affect *t*_i+1_** | | | | | |
| Intercept | 1.82 (0.20) |  |  |  | |
| Safety behaviours *t*_i_ | 0.07 (0.03) | [0.01; 0.12] | .07 | .014 | |
| Negative affect *t*_i_ | 0.26 (0.02) | [0.22; 0.31] | .27 | <.001 | |

*Note*. *b* = unstandardized coefficient. SE = standard error. CI = confidence interval. *β* = standardized coefficient. Each row segment represents a separate model. All models were calculated with fixed slopes and a random intercept.

**Supplementary Material 9**

**Table S-9**

*Multilevel Time-Lagged Effects in Daily Life With Fixed and Random Effect of Time Point i*

|  | ***b*(*SE*)** | **95%CI** | ***β*** | ***p*** | |
| --- | --- | --- | --- | --- | --- |
| **Outcome: Safety behaviours *t*_i+1_** | | | | |  |
| **Fixed Effects** | | | | |  |
| Intercept | 2.24 (0.22) |  |  |  | |
| Persecutory ideation *t*_i_ | 0.14 (0.02) | [0.09; 0.18] | .14 | <.001 | |
| Time point *i* | 0.00 (0.00) | [-0.00; 0.01] | .00 | .56 | |
| **Outcome: Safety behaviours *t*_i+1_** | | | | | |
| Intercept | 2.24 (0.22) |  |  |  | |
| Negative affect *t*_i_ | 0.10 (0.02) | [0.07; 0.14] | .09 | <.001 | |
| Time point *i* | 0.00 (0.00) | [-0.00; 0.01] | .00 | .55 | |
| **Outcome: Persecutory ideation *t*_i+1_** | | | | | |
| Intercept | 1.45 (0.25) |  |  |  | |
| Safety behaviours *t*_i_ | 0.13 (0.02) | [0.09; 0.18] | .12 | <.001 | |
| Time point *i* | 0.00 (0.00) | [-0.00; 0.00] | .00 | .66 | |
| **Outcome: Negative affect *t*_i+1_** | | | | | |
| Intercept | 1.79 (0.19) |  |  |  | |
| Safety behaviours *t*_i_ | 0.15 (0.3) | [0.10; 0.20] | .16 | <.001 | |
| Time point *i* | -0.00 (0.00) | [-0.01; 0.00] | .00 | .47 | |

*Note*. *b* = unstandardized coefficient. SE = standard error. CI = confidence interval. *β* = standardized coefficient. Each row segment represents a separate model. All models were calculated with fixed slopes, a random intercept and a random slope for time point *i*.

**Supplementary Material 10**

**Table S-10**

*Multilevel Time-Lagged Effects in Daily Life in Participants With ≥ 30% Response Rate (n = 57)*

| **Fixed Effects** | ***b*(*SE*)** | **95%CI** | ***β*** | ***p*** | |
| --- | --- | --- | --- | --- | --- |
| **Outcome: Safety behaviours *t*_i+1_** | | | | |  |
| Intercept | 2.28 (0.24) |  |  |  | |
| Persecutory ideation *t*_i_ | 0.14 (0.02) | [0.09; 0.18] | .15 | <.001 | |
| **Outcome: Safety behaviours *t*_i+1_** | | | | | |
| Intercept | 2.28 (0.24) |  |  |  | |
| Negative affect *t*_i_ | 0.12 (0.02) | [0.08; 0.16] | .11 | <.001 | |
| **Outcome: Persecutory ideation *t*_i+1_** | | | | | |
| Intercept | 1.54 (0.27) |  |  |  | |
| Safety behaviours *t*_i_ | 0.13 (0.02) | [0.08; 0.17] | .12 | <.001 | |
| **Outcome: Negative affect *t*_i+1_** | | | | | |
| Intercept | 1.72 (0.20) |  |  |  | |
| Safety behaviours *t*_i_ | 0.16 (0.03) | [0.11; 0.22] | .18 | <.001 | |

*Note*. *b* = unstandardized coefficient. SE = standard error. CI = confidence interval. *β* = standardized coefficient. Each row segment represents a separate model. All models were calculated with fixed slopes and a random intercept.

**Supplementary Material 11**

**Table S-11**

*Multilevel Time-Lagged Effects in Daily Life Using Multiple Imputation*

| **Fixed Effects** | ***b*(*SE*)** | **95%CI** | ***β*** | ***p*** | |
| --- | --- | --- | --- | --- | --- |
| **Outcome: Safety behaviours *t*_i+1_** | | | | |  |
| Intercept | 2.27 (0.23) |  |  |  | |
| Persecutory ideation *t*_i_ | 0.13 (0.02) | [0.09; 0.17] | .15 | <.001 | |
| **Outcome: Safety behaviours *t*_i+1_** | | | | | |
| Intercept | 2.27 (0.23) |  |  |  | |
| Negative affect *t*_i_ | 0.12 (0.02) | [0.08; 0.15] | .11 | <.001 | |
| **Outcome: Persecutory ideation *t*_i+1_** | | | | | |
| Intercept | 1.62 (0.26) |  |  |  | |
| Safety behaviours *t*_i_ | 0.13 (0.02) | [0.09; 0.18] | .13 | <.001 | |
| **Outcome: Negative affect *t*_i+1_** | | | | | |
| Intercept | 1.87 (0.20) |  |  |  | |
| Safety behaviours *t*_i_ | 0.17 (0.03) | [0.12; 0.22] | .19 | <.001 | |

*Note*. *b* = unstandardized coefficient. SE = standard error. CI = confidence interval. *β* = standardized coefficient. Each row segment represents a separate model. All models were calculated with fixed slopes and a random intercept. The imputation procedure is detailed in Supplementary Material 2.

**Supplementary Material 12**

**Table S-12**

*Multilevel Time-Lagged Effects in Daily Life in Participants With Severe or Very Severe Persecutory Ideation (n = 30)*

| **Fixed Effects** | ***b*(*SE*)** | **95%CI** | ***β*** | ***p*** | |
| --- | --- | --- | --- | --- | --- |
| **Outcome: Safety behaviours *t*_i+1_** | | | | |  |
| Intercept | 3.12 (0.36) |  |  |  | |
| Persecutory ideation *t*_i_ | 0.13 (0.03) | [0.08; 0.19] | .12 | <.001 | |
| **Outcome: Safety behaviours *t*_i+1_** | | | | | |
| Intercept | 3.11 (0.36) |  |  |  | |
| Negative affect *t*_i_ | 0.12 (0.03) | [0.07; 0.17] | .10 | <.001 | |
| **Outcome: Persecutory ideation *t*_i+1_** | | | | | |
| Intercept | 2.38 (0.43) |  |  |  | |
| Safety behaviours *t*_i_ | 0.14 (0.04) | [0.07; 0.21] | .13 | <.001 | |
| **Outcome: Negative affect *t*_i+1_** | | | | | |
| Intercept | 2.45 (0.31) |  |  |  | |
| Safety behaviours *t*_i_ | 0.16 (0.04) | [0.09; 0.24] | .17 | <.001 | |

*Note*. *b* = unstandardized coefficient. SE = standard error. CI = confidence interval. *β* = standardized coefficient. Each row segment represents a separate model. All models were calculated with fixed slopes and a random intercept.

**Supplementary Material 13**

**Table S-13**

*Multilevel Time-Lagged Effects in Daily Life With Random Slopes Added*

| **Fixed Effects** | ***b*(*SE*)** | **95%CI** | ***β*** | ***p*** | |
| --- | --- | --- | --- | --- | --- |
| **Outcome: Safety behaviours *t*_i+1_** | | | | |  |
| Intercept | 2.29 (0.23) |  |  |  | |
| Persecutory ideation *t*_i_ | 0.15 (0.03) | [0.10; 0.21] | .12 | <.001 | |
| **Outcome: Safety behaviours *t*_i+1_** | | | | | |
| Intercept | 2.29 (0.23) |  |  |  | |
| Negative affect *t*_i_ | 0.11 (0.02) | [0.07; 0.16] | .10 | <.001 | |
| **Outcome: Persecutory ideation *t*_i+1_** | | | | | |
| Intercept | 1.40 (0.23) |  |  |  | |
| Safety behaviours *t*_i_ | 0.17 (0.04) | [0.09; 0.26] | .13 | <.001 | |
| **Outcome: Negative affect *t*_i+1_** | | | | | |
| Intercept | 1.67 (0.19) |  |  |  | |
| Safety behaviours *t*_i_ | 0.17 (0.05) | [0.08; 0.26] | .17 | <.001 | |

*Note*. *b* = unstandardized coefficient. SE = standard error. CI = confidence interval. *β* = standardized coefficient. Each row segment represents a separate model. All models were calculated with fixed and random slopes, and a random intercept.
